# Buffer wards for the control of COVID‐19 transmission in hospitals

**DOI:** 10.1002/ctm2.223

**Published:** 2020-11-08

**Authors:** Wensi Zhao, Yi Gao, Qiuni Xu, Wanjun Ding, Dedong Cao, Zhuya Xiao, Jiayu Chen, Li Yan, Chen Zhao, Xiaoxu Li, Ying Chen, Qian Chen, Yongshun Chen

**Affiliations:** ^1^ Department of Clinical Oncology Renmin Hospital of Wuhan University Wuhan Hubei China; ^2^ Department of Hematology Renmin Hospital of Wuhan University Wuhan Hubei China; ^3^ Department of Health Check Center Renmin Hospital of Wuhan University Wuhan Hubei China

To the Editor:

The outbreak of the coronavirus disease 2019 (COVID‐19) has brought great challenges to the routine diagnosis and treatment of patients.^1^ It has been proved in our clinical practice that the buffer ward, as the intermediate platform of pre‐examination and risk screening for patients requiring hospitalization, was an effective way to control the COVID‐19 transmission in hospitals.^2^ However, limited literatures reported the operation and efficiency of the buffer ward. We therefore summarized the admission, characteristics, and outcomes of the patients in buffer wards in our hospital.

A total of 1003 patients were included (median age 57 years [interquartile range, IQR, 48–65; range 2–95 years]; 49.5% female; 36.0% cancer) between March 11 and April 23, 2020 (Table 1). Cancer patients, who were vulnerable to COVID‐19, were also the focus of this study. Subgroup analyses were performed between cancer and noncancer patients. The demographic distribution between two groups was well balanced (median age 57 years [IQR 50–64; range 3–88 years] and 51.3% female vs median age 57 years [IQR 47–67; range 2–95 years] and 48.4% female; *P *= .68 for age and *P *= .39 for gender). Among all the 361 cancer patients, those with thoracic tumors (98, 27.2%), mainly lung cancer, have the most urgent need for hospitalization, followed by gastrointestinal tumors (67, 18.6%) and breast cancer (56, 15.5%) (Table 1). Among the 642 noncancer patients, those with chronic cardio‐cerebrovascular diseases (115, 31.2%) were the most affected population, which mirrors findings of other literatures.^3^, ^4^ In addition, 19 (3.0%) patients were admitted for thrombotic disease, reflecting the inevitable reality of limited social activities under the epidemic.^5^

**TABLE 1:** Baseline characteristics of patients hospitalized in buffer wards

Clinical characteristics	Number (%)
Total number	1003
Age (years), median (IQR) [range]	57 (48‐65) [2‐95]
Sex	
Female	496 (49.5)
Male	507 (50.5)
Cancer	361 (36.0)
Age (years), median (IQR) [range]	57 (50‐64) [3‐88]
Sex	
Female	185 (51.3)
Male	176 (48.8)
Subcategories	
Head and neck cancer	38 (10.5)
Thoracic cancer	98 (27.2)
Digestive cancer	67 (18.6)
Breast cancer	56 (15.5)
Female genital cancer	49 (13.6)
Male genitourinary cancer	17 (4.7)
Lymphatic hematopoietic cancer	28 (7.8)
Endocrine cancer	3 (0.8)
Bone and soft tissue cancer	3 (0.8)
Skin cancer	2 (0.6)
Noncancer	642 (64.0)
Age (years), median (IQR) [range]	57 (47‐67) [2‐95]
Sex	
Female	311 (48.4)
Male	331 (51.6)
Subcategories	
Cardiovascular disease	75 (11.7)
Respiratory disease	23 (3.6)
Digestive disease	61 (9.5)
Hematological disease	21 (3.3)
Breast and thoracic surgery disease	40 (6.2)
Cerebrovascular and neurological disease	125 (19.5)
Interventional and vascular surgery disease	19 (3.0)
Head and neck and otolaryngological disease	17 (2.7)
Kidney disease^a^	62 (9.7)
Urologic disease	59 (9.2)
Liver disease^b^	12 (1.9)
Orthopedic disease	47 (7.3)
Ophthalmic disease	36 (5.6)
Gynecological disease	35 (5.5)
Metabolic disease	6 (0.9)
Psychiatric disease	4 (0.6)

*Note*. Data are presented as absolute numbers and percentages (%) or median (IQR) [range]. *χ* ^2^ test and one‐way ANOVA were used for comparative analyses between the two subgroups of patients in terms of sex and age, respectively, by SPSS24.0 software.

Abbreviation: IQR, interquartile range.

^a^ Assessed based on a diagnosis of acute and chronic kidney disease in medical history by International Statistical Classification of Diseases and Related Health Problems, 11th Revision (ICD‐11) coding.

^b^ Assessed based on a diagnosis of infectious, drug‐induced, or toxic liver disease in medical history by ICD‐11 coding.

The process of hospitalization and risk stratification of COVID‐19 is shown in Figure 1. All patients would undergo two rounds of risk screening in outpatient and emergency department and buffer ward, respectively. Patients with confirmed infection would be reported immediately and sent to designated hospitals, meanwhile the suspicious close contacts would be isolated for another 14‐day quarantine.

**FIGURE 1:**
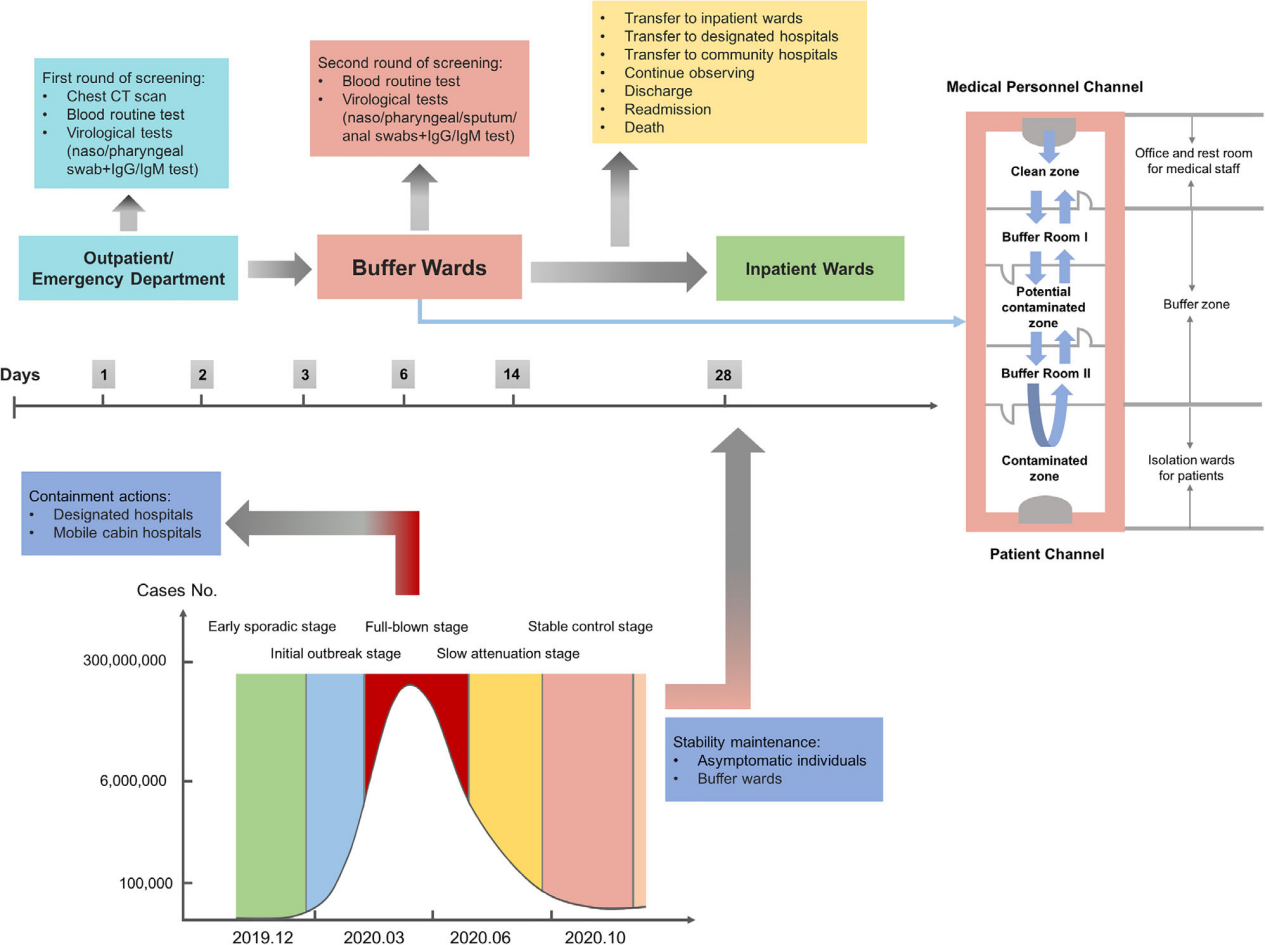
Hospitalization and risk stratification strategy of COVID‐19 for patients during the epidemic remission stage. At the beginning of the outbreak, the epidemic was controlled mainly through designated hospitals and mobile cabin hospitals, whereas in the remission stage, the main strategy was to maintain stability by screening of asymptomatic individuals and setting up buffer wards. The first round of risk screening in outpatient or emergency department includes chest CT scan, blood routine, and virological tests (nucleic acid test of nasopharyngeal and oropharyngeal swab and serological test of IgM and IgG antibody). Unconfirmed asymptomatic patients were temporarily transferred to buffer ward for a second round of screening, including blood routine, nucleic acid tests of nasopharyngeal, oropharyngeal, sputum, and anal swabs, and serological tests of IgM and IgG antibody. Other specialized examinations may also be conducted if necessary, such as cardiac troponin I (cTnI) and craniocerebral CT scan. The three zones and two channels of buffer wards refer to the clean zone, potential contaminated zone, contaminated zone, medical personnel channel, and patient channel. The buffer wards were classified into subspecialties: (1) comprehensive surgical buffer ward, (2) comprehensive internal medicine buffer ward, (3) buffer ward for breast, thyroid and reproductive system diseases, (4) buffer ward for gastrointestinal bleeding or other emergencies, (5) oncology and hematology buffer ward, (6) ophthalmic buffer ward, (7) psychiatric buffer ward, and so on

As an important gateway to control the epidemic in hospitals, the buffer ward was temporarily constructed based on the principle of three zones and two channels, and was under closed‐end management to reduce the nosocomial cross‐infection (Figure 1). Specialist consultation became the bridge of communication. A standard two‐ or six‐occupant ward can only accommodate a maximum of one or two patients, respectively, and those previously infected patients must be admitted to a separate ward. To improve efficiency and save medical resources, we classified buffer wards into subspecialties for centralized management of similar patients.

It was reported that the viral load of asymptomatic patients was no less than that of symptomatic patients.^8^ Close monitoring and preventing them from gathering remains the priority. Three months ago, the Wuhan Health Committee had organized the nucleic acid test of COVID‐19 for nearly 10 million residents, and only 300 (0.003%) asymptomatic infected individuals were eventually detected. In our study, the asymptomatic infection rate of hospitalized patients was relatively higher, at 3.8% (Table 2). And of the 38 asymptomatic infected patients, two developed a confirmed infection. Among all admitted patients, only one newly confirmed and one re‐positive cases were found. Of all the nine close contacts, none has developed symptoms or confirmed to be infected after rigorous medical observation (Table 2). Therefore, despite relatively high density of asymptomatic individuals in hospitals, the transmission of the virus was effectively blocked, and that is maybe what the buffer wards were for.

**TABLE 2:** Laboratory and virological analysis of patients hospitalized in buffer wards

	No. (%)			
Indicators	Cancer patients	Noncancer patients	Reference ranges	*P*‐value
Temperature, >37.3°C	5 (1.4)	24 (3.7)		.03^a^
Chest CT, viral pneumonia imaging	0 (0.0)	5 (0.8)		.09
Length of stay,^a^ >3 days	33 (9.1)	39 (6.1)		.07
Lymphopenia, <1.1 × 10^9^/L	115 (31.9)	156 (24.5)		.01^a^
Laboratory indicators, median (IQR) [No.]				
WBC (absolute count, × 10^9^/L)	6.1 (4.8‐7.9) [361]	6.6 (5.2‐8.4) [639]	3.5‐9.5	>.99
NEU	3.8 (2.8‐5.5) [361]	4.3 (3.1‐5.8) [636]	1.8‐6.3	>.99
NEU%	65.6 (57.6‐73.1) [361]	65.7 (57.0‐73.5) [636]	40‐75	>.99
LYM	1.4 (1.0‐1.8) [361]	1.5 (1.1‐2.0) [638]	1.1‐3.2	.37
LYM%	23.7 (16.5‐30.4) [361]	23.8 (15.7‐31.6) [636]	20‐50	>.99
MON	0.5 (0.4‐0.7) [361]	0.50 (0.4‐0.7) [636]	0.1‐0.6	>.99
PLT	211 (155‐259) [361]	211.5 (167‐267.3) [636]	125‐350	>.99
NLR (%)	2.8 (1.9‐4.4) [361]	2.8 (1.8‐4.7) [636]	0.5‐5.7	>.99
LMR (%)	2.8 (1.9‐4.0) [361]	3.0 (1.9‐4.3) [636]	1.8‐32	>.99
hsCRP (mg/L)	2.7 (0.5‐27.2) [305]	2.4 (0.4‐16.8) [571]	0‐5	>.99
LDH (U/L)	207 (170‐262.5) [284]	201.5 (166‐249.8) [521]	120‐250	.08
PCT (ng/mL)	0.1 (0.0‐0.7) [61]	0.2 (0.1‐0.8) [125]	<0.1	>.99
Virological indicators				
Previous infection	3 (0.8)	3 (0.5)		.47
Hospitalized this time				
Nucleic acid positive	7 (1.9)	6 (0.9)		.18
IgM positive	3 (0.8)	4 (0.6)		.70
IgG positive	8 (2.2)	17 (2.6)		.67
Re‐positive	1 (0.3)	0 (0.0)		.18
Confirmed infection^b^	0 (0.0)	1 (0.2)		.45
Asymptomatic infection	16 (4.4)	22 (3.4)		.42
Asymptomatic to confirmed infected	1 (0.3)	1 (0.2)		.68
Close contacts^c^	5 (1.4)	4 (0.6)		.22
Confirmed infection in close contacts	0 (0.0)	0 (0.0)		–

*Note*. Data are presented as median (IQR) [No.] or absolute numbers and percentages (%), where No. is the total number of patients with available data. Multiple *t*‐test and *χ* ^2^ test were used for comparative analysis by SPSS24.0 software.

Abbreviations: CT, computed tomography; hsCRP, hypersensitive C‐reactive protein; IQR, interquartile range; LDH, lactate dehydrogenase; LMR, lymphocyte‐to‐monocyte ratio; LYM, lymphocyte; MON, monocyte; NEU, neutrophil; NLR, neutrophil‐to‐lymphocyte ratio; PCT, procalcitonin; PLT, platelet; WBC, white blood cell.

^a^ Length of stay begins with admission to the buffer ward time and ends with transfer to an inpatient ward time, transfer to a designated hospital time, discharge time, or time at death. It does not include time in the outpatient or emergency department.

^b^ Confirmed cases were diagnosed according to the Guidelines for the Diagnosis and Treatment of COVID‐19 by the National Health Commission (trial version 7).

^c^ Close contacts were determined according to the close contacts management protocol of Diagnosis and Treatment Plan for COVID‐19 (trial version 6).

^*^ *P *< .05 was considered statistically significant.

As expected, most patients were transferred to inpatient wards after 3 days of transition in buffer wards, three patients were transferred to designated hospitals, one was transferred to community hospital, three were readmitted, 12 were discharged, and six died.

Fever and cough are the most typical clinical manifestations of COVID‐19, while shortness of breath, sore throat, nasal congestion, diarrhea, nausea, vomiting, fatigue, anorexia, headache, myalgias, dysosmia, dysgeusia, hearing loss, ischemic and hemorrhagic stroke, encephalopathy, encephalitis, and thyroid inflammation are other atypical symptoms that should also be taken seriously. Here, lymphopenia and fever were two statistically significant indicators between cancer and noncancer patients (*P *= .01, 31.9% vs 24.5% and *P *= .03, 1.4% vs 3.7%; Table 2). The former may be associated with the immunosuppressive status in cancer patients, whereas the latter may be related to acute abdomen such as pancreatitis and appendicitis in noncancer patients.^6^, ^7^ But what is interesting to note here is that cancer patients in buffer wards did not show a higher risk of infection (*P *= .45, 0% vs 0.2%), indicating that immunodeficiency may not be the only susceptibility factor for them, and that active hospitalization for primary disease was feasible under the current objective conditions.

A recent analysis showed that 22 million cancer screenings may have been canceled or delayed between March and June 2020, which directly contributed to 80 000 missed cancer cases.^9^ Therefore, with hospital's prevention and control measures in place, we encourage cancer patients to overcome panic caused by COVID‐19 and actively seek medical treatment under the premise of adequate self‐protection. For the general population, it is also recommended to face the need of seeking care and put the routine cancer screening on the agenda.

As a model hospital in Hubei province, our hospital had a good representation of patients and the management of buffer wards was also a typical demonstration. But, as a single‐center retrospective study, the interpretation of findings may be limited by the observational design.

In conclusion, this is the first dedicated case series observing patients who were admitted to buffer wards during the epidemic remission stage. Our data suggest that for patients requiring hospitalization, especially cancer patients, the buffer wards can effectively cut off the route of transmission, classify asymptomatic cases, and control the spread of COVID‐19. It offers useful practical experience to others globally to fight against the pandemic. In short, we advocate setting up buffer wards wherever possible on the basis of the principle of “three zones and two channels” and closed‐end management, but it also depends on various external conditions.

## ACKOWLEDGMENTS

This work was supported by the National Natural Science Foundation of China (No. U1604175 to YC) and Fundamental Research Funds for the Central Universities of China (2042020kf0110 to Wensi Zhao). We thank all the patients involved in the study.

## CONFLICT OF INTEREST

The authors declare that there is no conflict of interest.

## ETHICS STATEMENT AND CONSENT TO PARTICIPATE

This study was approved by the Medical Ethics Committee of Renmin Hospital of Wuhan University. All the patients gave their written informed consent in accordance with the Declaration of Helsinki. The authors have obtained consent from the participants to publish/report individual patient data.

## FUNDING INFORMATION

National Natural Science Foundation of China, Grant Number: U1604175; Fundamental Research Funds for the Central Universities of China, Grant Number: 2042020kf0110

## AUTHOR CONTRIBUTIONS

Yongshun Chen and Qian Chen conceived and designed the clinical study; Wanjun Ding and Dedong Cao conducted quality control and supervision; Wensi Zhao, Yi Gao, Zhuya Xiao, Jayu Chen, Li Yan, and Chen Zhao performed statistical analyses; Wensi Zhao and Yongshun Chen drafted and revised the manuscript; all the authors collected clinical data, analyzed, discussed, and revised the important intellectual content of the manuscript.

## Data Availability

The published data are available upon request from the corresponding authors.
